# Combination of transcatheter arterial chemoembolization and CT-guided percutaneous segment ablation for hepatocellular carcinoma therapy: A retrospective study

**DOI:** 10.1097/MD.0000000000005422

**Published:** 2016-11-28

**Authors:** Wei Li, Yang Wang, Wenfeng Gao, Jiasheng Zheng

**Affiliations:** Center of Interventional Oncology and Liver Diseases, Beijing You’an Hospital, Capital Medical University, Beijing, P.R. China.

**Keywords:** ablation, hepatocellular carcinoma, percutaneous, segment, survival

## Abstract

Treatment option for liver cancer patients with large tumor >5 cm and/or portal vein tumor thrombosis is very limited. New treatment strategy is badly needed. Our study is to determine the safety and treatment efficacy of a new minimally invasive treatment strategy—liver segment thermal ablation.

Late-stage hepatocellular carcinoma patients were included and treated with percutaneous ablation to destroy the entire tumor-containing liver segment to reduce reoccurrence and prolong survival. Transcatheter arterial chemoembolization was used before ablation to label tumor margin. The patients were followed up routinely.

The patients were followed up for 8 to 95 months. Mean overall survival (OS) (n = 6) was 21.5 months (range 8–95). For patients in BCLC stage B (n = 2), average OS was 16 months; for those in stage C (n = 4), mean OS was 25 months (range 15–95). Out of all 6 patients, 2 reoccurred within 1 year, and 1 reoccurred after 13 months postoperatively. The average alpha-fetoprotein was dropped from 1153.69 to 41.22 μg/L postoperatively. No severe intra or postoperative complications were observed.

Our preliminary data indicated that transcatheter arterial chemoembolization + segment ablation is safe and benefits survival significantly for late-stage hepatocellular carcinoma patients. A prospective multicenter, randomized trial comparing focal and segment ablation is now ongoing in China (Trial Registry Number, ChiCTR-TRC-12002786).

## 1 Introduction

Hepatocellular carcinoma (HCC) is one of the most common and fatal malignancies.^[1]^ It is difficult to control because of no effective systemic treatment. Many approaches have been tried and their therapeutic effects are still far away from ideal.^[2,3]^ Surgical resection sustains intensive local injury, a high reoccurrence rate, and does not benefit overall survival (OS) for tumor ≥30 mm.^[1,4–6]^ Mortality is particularly high for those with a tumor ≥50 mm since they are not fit for liver transplantation or resection.^[7,8]^ Percutaneous ablation has the advantage of being minimally invasive and could be used for almost all HCC patients. However, recurrence of patients with a tumor ≥50 mm was high after ablation.^[9–12]^ Transcatheter arterial chemoembolization (TACE) is the only option offered by the guideline for these patients. Although TACE is effective to label tumors, it is a palliative measure with poor treatment efficacy.^[13]^ Patients with portal vein tumor thrombosis (PVTT) were found in 10% to 40% of HCC patients at diagnosis, and suffered a dismal OS of 8.1 to 16.9 months after treatment.^[8,9,14]^ Those with tumor thrombosis at main portal vein (MPVTT) only demonstrated around half of above OS.^[15]^ The only treatment option for HCC + PVTT patients is sorafenib as per Barcelona guideline.^[8]^ However, sorafenib extends patient life span for only 2.7 months in average.^[4]^ New treatment strategy is badly needed for patients with a tumor ≥50 mm and/or PVTT, which, unfortunately, accounts for around 50% of all HCC patients.^[14]^

Here, we tried to use TACE with iodized oil to label the tumors and after percutaneous ablation to destroy the entire tumor-containing segment to reduce reoccurrence and prolong survival.

## 2 Materials and methods

### 2.1 Patients

Between March 2008 and May 2015, we performed TACE + segment ablation on 6 HCC patients who received no prior treatment, except 2 treated with surgical resection and reoccurred. The inclusion criteria were as follows: HCC diagnosed by pathology or typical image; tumor diameter >50 mm or PVTT(+); Child-Pugh A. The exclusion criteria were as follows: severe underlying illness; platelet count <50 × 10^3^/μL; number of tumor nodules >5; extrahepatic metastasis; patient refusal.

### 2.2 Ethical approval

Beijing You’an Hospital Ethics Committee has approved this study. It covered the data analysis of all patients. All involving subjects had given a written informed consent upon admission for their information to be stored and used for research. Human experimentation guidelines of P.R. China were followed.

### 2.3 Transcatheter arterial chemoembolization

Transcatheter arterial chemoembolization was performed in all patients by senior interventional radiologists. After the introduction of a 5-F pigtail catheter through the femoral artery under local anesthesia, an angiographic survey of the main abdominal vessels was performed. Superselective cannulation of artery supplying the tumor was performed as necessary. Depending on the size and arterial supply of the tumor, 2 to 15 mL of iodized oil (Lipiodol; Huaihai Pharmaceutical Factory, Shanghai, China) was slowly injected with fluoroscopic guidance. Ablation was performed 1 to 2 weeks after TACE.

### 2.4 Percutaneous thermal ablation

Pethidine 50 mg and promethazine 25 mg were given intravenously as basal anesthesia before operation. All ablative procedures were done under local anesthesia with 1% lidocaine. Real-time guidance of the ablation procedures was by intraoperative computed tomography (CT) scans (Toshiba, Tokyo, Japan).

The microwave ablation (MWA) system (Qinghai Ltd., Nanjing, P.R. China) and radiofrequency ablation (RFA) system (Covidien, Shanghai, P.R. China or RITA Medical Systems, Mountain View, CA) were used in this study. For MWA, the Qinghai generator provided a frequency of 2450 MHz and a power output of 10 to 80 W. For RFA, a generator with a frequency of 290 kHz and maximum power output of 200 W was used. The shafts of all antennas were cooled by 4°C, circulating saline solution driven by a pump continuously through dual channels inside the antenna shaft.

### 2.5 Follow-up

All patients were followed up routinely at every month within the first 3 months after operation, then every 3 months. Complete blood count, liver function tests, urine tests, coagulation function, serum tumor markers, quantitative hepatitis B virus (HBV)-DNA and contrast-enhanced abdominal CT were performed on each follow-up. No patient was lost from follow-up.

## 3 Results

All 6 HCC patients were HBV-infected, and 5 were male. The age ranged from 39 to 68 years. Four patients were in Bacelona Clinic Liver Cancer (BCLC) stage C and 2 in stage B. Two had portal vein hypertension, and 5 had cirrhosis. No one had diabetes or coronary artery diseases, and 2 were with hypertension, which was well-controlled. Details of the 6 patients are listed in Table 1.

**Table 1:**
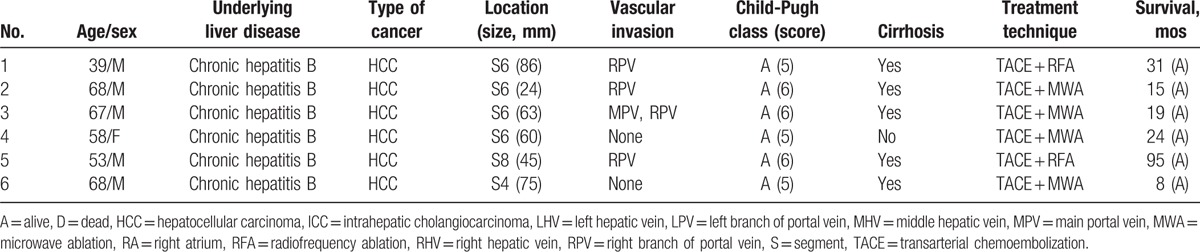
Patient characteristics.

Total number of tumor nodules was 9. Five patients had solitary tumor nodule and 1 patient had 4 tumor nodules. The tumor diameter ranged from 24 to 86 mm. Four patients had PVTT, 1 of which suffered MPVTT. The average preoperative alpha-fetoprotein (AFP) was 1153.69 ug/L.

During ablation, pethidine 50 mg and promethazine 25 mg were given intravenously as basal anesthesia before operation. No systemic anesthesia was applied. All ablative procedures were done under local anesthesia with 1% lidocaine. Real-time guidance was by intraoperative CT scans. Four patients were treated with MWA and 2 with RFA. Percutaneous ablation was performed on 4 patients at segment VI, 1 at segment IV, and 1 at segment VIII. There were 2 patients who received 2 sequential operations to complete segment ablation, respectively. The other 4 were done at one time. Intraoperative complications included transit hypertension, mild hepatalgia, nausea, and chest pain, all of which were resolved soon. No severe intra or postoperative complications were observed. One patient had shoulder pain at day 1 postoperatively, and 1 had hepatalgia and nausea at day 2. Transit liver function abnormality was observed in all patients and was resolved quickly.

All patients were followed up routinely. The follow-up period ranged from 8 to 95 months (median 21.5). Five patients (83.3%) were followed up for >1 year, 3 (50.0%) for >2 years, 1 (16.7%) for >3 years, and 1 (16.7%) for >5 years.

**Figure 1:**
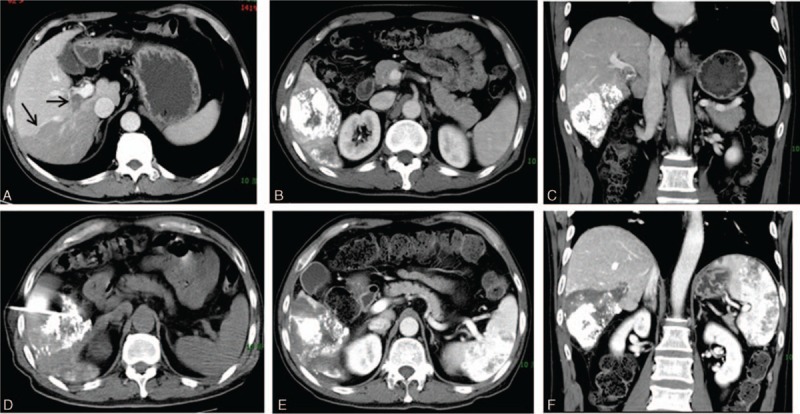
Computed tomography (CT) scanning of a patient treated by CT-guided percutaneous segment VI ablation therapy. (A) Preoperative contrast-enhanced CT scan of the liver tumor mass. Black arrow: tumor mass and tumor thrombus in portal vein. (B, C) CT scanning images after TACE. The tumor mass was labeled by iodized oil injected via TACE. (D) Intraoperative CT scan of the ablation against segment VI. (E, F) Postoperative contrast-enhanced CT scan at 1 month after the ablation therapy. The tumor-containing segment VI was completed ablated.

The average postoperative AFP was 41.22 μg/L. Overall median OS (MOS) was 21.5 months (range 8–95). For patients in BCLC stage B (n = 2), average OS was 16 months, and for those in stage C (n = 4), MOS was 25 months (range 15–95).Three patients suffered reoccurrence. Two patients reoccurred within 1 year and 1 reoccurred after 13 months postoperatively. The one with multiple tumor nodules reoccurred after 6 months, and 1 in 2 months (in a non-neighboring liver segment). They were treated with ablation again. All of them had PVTT or MPVTT. CT scans of a typical case, shown in Fig. 1.

## 4 Discussion

In HCC therapy, how to treat large tumor or PVTT and how much margin is safe to prevent reoccurrence are always in great debate. Currently, there is on satisfactory treatment for these patients. TACE and percutaneous thermal ablation are minimally invasive and even without the need for systemic anesthesia. They are promising for patients with advanced HCC. However, the reported reoccurrence was high.^[9,10,16]^ Traditionally, ablation therapy only destroys the tumor area with a 0.5 to 1.0 cm margin. However, increased tumor size parallels the risk of vascular invasion and dissemination along blood flow distribution.^[8,9]^ Therefore, we hypothesized that extensive ablation against the entire tumor-containing segment may reduce reoccurrence and prolong survival. TACE was used to mark tumor margins, PVTT, and possible dissemination, before segment ablation. We believe that we are the first to try and evaluate this treatment strategy.

Current publications indicated that MOS for BCLC class B and C patients was 20 and 11 months, respectively.^[14]^ Our data provided a MOS of 21.5 months (range 8–95), and a MOS of 25 months (range 15–95) for patients in BCLC stage C. The best reported OS for HCC + PVTT and HCC + MPVTT were 8.1 to 16.9 months and 4.4 to 9.9 months.^[7,14,17]^ In our study, they were 25 and 19 months, respectively. And no one has died yet. No severe complications of TACE or percutaneous ablation reported in the past^[18,19]^ were observed in the study. Significant improvement has been observed, although further study with more study subjects is warranted.

The present study has its limitations. First of all, the sample size was small. Second, there was no control group to compare with. A prospective, multicenter, randomized controlled trial comparing focal and segment ablation is now ongoing in China (Trial Registry Number, ChiCTR-TRC-12002786).

In conclusion, our preliminary data indicated that TACE + segment ablation is safe and can prolong OS significantly.

## 5 Summary statement

A new ablation therapy strategy, percutaneous segment ablation, was tried on 6 advanced HCC patients. TACE was used to label tumor margins before ablation. No severe intra- or post-operative complications were observed. The patients were followed up and the overall survival was successfully extended.

Advances in knowledge:

1. Segment ablation could be a safe and effective treatment strategy to treat advanced HCC patients.
2. Minimally invasive percutaneous ablation therapy could be successfully used to treat advanced stage HCC with portal vein tumor thrombosis.
3. Transcatheter arterial chemoembolization could be helpful before ablation therapy to label tumor margins.

Implications for patient care:

1. Patients with advanced hepatocellular carcinoma could be treated effectively by percutaneous segment ablation therapy instead of only relying on sorafenib according to current guideline.
2. In hepatocellular carcinoma therapy, percutaneous ablation has become more and more important, including for patients at advanced stage
3. For advanced HCC patients who cannot accept surgery or general anesthesia, percutaneous ablation is a good option.
